# Pulmonary Nodule Detection and Classification Using All-Optical Deep Diffractive Neural Network

**DOI:** 10.3390/life13051148

**Published:** 2023-05-09

**Authors:** Junjie Shao, Lingxiao Zhou, Sze Yan Fion Yeung, Ting Lei, Wanlong Zhang, Xiaocong Yuan

**Affiliations:** 1 Nanophotonics Research Center, Institute of Microscale Optoelectronics, Shenzhen University, Shenzhen 518060, China; 2 State Key Laboratory on Advanced Displays and Optoelectronics Technologies, Department of Electronic & Computer Engineering, Hong Kong University of Science and Technology, Hong Kong SAR, China; 3 Research Center for Humanoid Sensing, Research Institute of Intelligent Sensing, Zhejiang Lab, Hangzhou 311100, China

**Keywords:** pulmonary nodules, all optical, deep diffractive neural network, aided diagnosis, real time

## Abstract

A deep diffractive neural network (D2NN) is a fast optical computing structure that has been widely used in image classification, logical operations, and other fields. Computed tomography (CT) imaging is a reliable method for detecting and analyzing pulmonary nodules. In this paper, we propose using an all-optical D2NN for pulmonary nodule detection and classification based on CT imaging for lung cancer. The network was trained based on the LIDC-IDRI dataset, and the performance was evaluated on a test set. For pulmonary nodule detection, the existence of nodules scanned from CT images were estimated with two-class classification based on the network, achieving a recall rate of 91.08% from the test set. For pulmonary nodule classification, benign and malignant nodules were also classified with two-class classification with an accuracy of 76.77% and an area under the curve (AUC) value of 0.8292. Our numerical simulations show the possibility of using optical neural networks for fast medical image processing and aided diagnosis.

## 1. Introduction

Artificial intelligence has become a highly researched and widely discussed topic in recent years. Deep neural networks have been utilized to solve various tasks such as natural language processing [1,2,3], image classification [4,5,6], object detection [7,8,9,10], semantic segmentation [11,12,13], etc. As the complexity and size of deep neural networks increase, more parameters need to be computed, which requires more time to process the input data. However, real-time processing tasks such as autonomous driving [14,15] are highly demanded, presenting a challenge to traditional parallel computing devices, e.g., graphics processing units (GPUs). Despite significant advantages in GPU technology in recent years, it is increasingly difficult to achieve further developments with silicon-based processing technology.

Optical neural networks represent a new and exciting direction in deep learning architecture, utilizing the propagation of light waves and modulation of the light field with optical devices to achieve ultra-fast computational speeds. Recent research has proposed various structures, including optical convolution networks [16,17], Mach–Zehnder interferometer-based optical networks [18,19,20], optical spiking neural networks [21,22], and diffractive deep neural networks (D2NNs) [23,24,25,26,27,28,29,30,31,32,33,34,35,36,37]. Due to D2NNs’ simple structure with high parallel operation and low cost, there has been significant interest in D2NN research over the past few years, including increasing the networks’ computation ability [24,25,26,27,28,29] and robustness [30,31,32]. For instance, Li et al., proposed a differential diffractive network to enhance classification accuracy [24]. Zhou et al., used multiple photoelectric and electro-optic conversions to provide non-linear computation and improve the network’s inferential ability [26]. Moreover, various problems have been solved by D2NNs, such as image classification [23,33], filtering [34,35], logical operations [36,37], object detection [25,26], etc.

Medical imaging provides images of the human body’s internal organs. The image processing plays a crucial role in diagnosing various diseases. Computed tomography (CT) imaging is one of the best methods for detecting and analyzing pulmonary nodules. In recent years, deep learning has been applied to CT image analysis, including pulmonary nodule detection [38,39,40] and classification [39,41,42], demonstrating its effectiveness in this field. Experiments using D2NNs have been discussed in many research papers. Lin et al., reported that a 3D-printed optical diffractive layer can modulate a Terahertz light source and be composed into all-optical D2NNs, which were further applied for MNIST and Fashion-MNIST datasets [23]. Chen et al., presented optical diffractive units for visible wavelengths fabricated by a multi-step photolithography–etching method [43]. Luo et al., showed that the optical diffractive layers could be fabricated with a metasurface structure with 400 nm diffractive units to modulate visible light [44]. These studies demonstrated the feasibility of using all-optical networks in experiments.

In this paper, we propose the use of an all-optical D2NN for pulmonary nodule detection and classification to increase the speed of image processing and reduce waiting times for patients. The Lung Image Database Consortium image collection (LIDC-IDRI) dataset was used to train and test the network. In the numerical simulation, pulmonary nodule detection achieved a recall rate of 90.47% through the classification of whether pulmonary nodules existed or not. Using the trained all-optical network, slices of lung CT images were scanned to obtain information on the pulmonary nodules’ positions. For the pulmonary nodule classification, the network was adopted to classify benign and malignant nodules, achieving an accuracy of 76.77% and an area under the curve (AUC) of 0.8292, indicating the possibility of using all-optical neural networks in medical image processing. Furthermore, by combining the network with optical non-linear materials for advanced computation, the computing power and accuracy of the network can be further improved, indicating the possible uses of all-optical D2NNs in fast medical-image-aided diagnosis.

## 2. Methods

### 2.1. Dataset

The LIDC-IDRI dataset is a well-known database of thoracic CT scans and diagnostic results related to lung cancer [41,42]. In this dataset, 4 experienced thoracic radiologists analyzed the details of pulmonary nodules to classify them into 5 categories, with higher numbers indicating more serious nodules diagnosis. All the information were recorded in the XML files. In this work, the CT scans’ XML files were used to locate the pulmonary nodules and extract their diagnostic results.

### 2.2. All-Optical Diffractive Deep Neural Network

**Figure 1:**
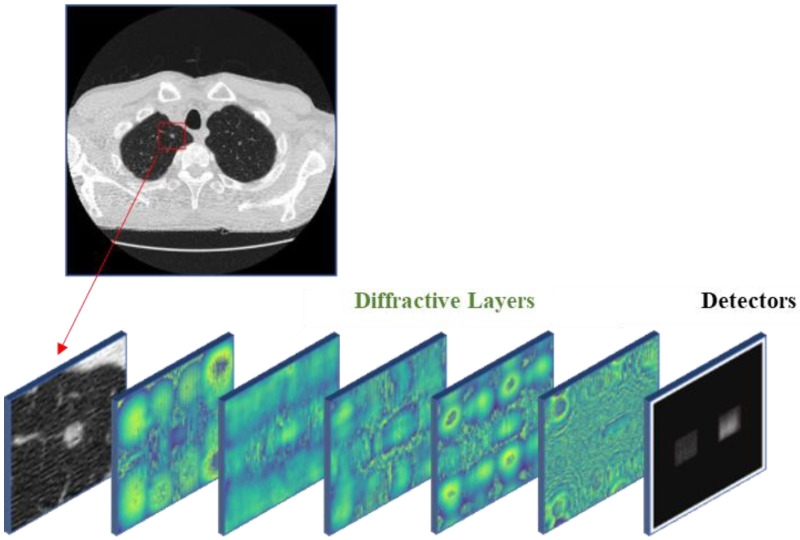
Schematic diagram of all-optical D2NN. The CT image, which serves as the input of the network, is clipped to a size of 50 × 50 pixels (as shown in red square region) and modulated for the amplitude of the light field. The detectors at the designed positions record the intensity of the output field.

An all-optical D2NN is a novel approach that combines multiple optical diffractive layers, as illustrated in Figure 1. In this approach, the light field propagates in free space, and its phase and amplitude are modulated by a diffractive device, such as a spatial light modulator (SLM), liquid-crystal volume phase plates [45], or 3D-printed layers. According to the Huygens–Fresnel principle [46], the light field can be considered as many secondary wave sources, and the resulting propagation can be computed by the envelope influence of all secondary waves. The propagation of the secondary wave can be computed using scalar diffraction theory, such as the Rayleigh–Sommerfeld diffraction theory [46], and the impulse response can be expressed as:

(1) $$w_{i}^{l}\left(x,y,z\right)=\frac{1}{2\pi }\frac{z-z_{i}}{r}\left(\frac{1}{r}-jk\right)\frac{\exp(jkr)}{r},\;$$

where k=2π/λ is the wave vector, λ is the wavelength, i refers to the i-th neurons in the l-th layer, and the distance between the current neuron and the i-th neuron in the l-th layer is given by $r=\sqrt{{\left(x-x_{i}\right)}^{2}+{\left(y-y_{i}\right)}^{2}+{\left(z-z_{i}\right)}^{2}}$. The imaginary unit is represented by $j=\sqrt{-1}$. The diffractive layer modulation can be expressed as $t_{i}^{l}\left(x_{i},y_{i},z_{i}\right)=a_{i}^{l}\left(x_{i},y_{i},z_{i}\right)\exp(j\varphi_{i}^{l}\left(x_{i},y_{i},z_{i}\right))$, where $a_{i}^{l}\left(x_{i},y_{i},z_{i}\right)$ and $\varphi_{i}^{l}\left(x_{i},y_{i},z_{i}\right)$ represent the amplitude and phase modulation factors, respectively. These factors are considered as trainable parameters in the neural network. The light field $u_{i}^{l}\left(x_{i,}y_{i},z_{i}\right)$ in the i-th neurons in the l-th layer can be express as:

(2) $$u_{i}^{l}\left(x_{i,}y_{i},z_{i}\right)=t_{i}^{l}\left(x_{i,}y_{i},z_{i}\right)\cdot \sum_{k}u_{k}^{l-1}w_{k}^{l-1},$$

The angular spectrum method used in training the networks describes light field propagation in Fourier space, reducing the operation time in the training section [46]. Thus, Equation (2) can be written as follows:

(3) $$u_{i}^{l}\left(x_{i,}y_{i},z_{i}\right)=t_{i}^{l}\left(x_{i,}y_{i},z_{i}\right)\cdot F^{-1}\left(U^{l-1}\left(u,v\right)\exp(j2\pi \gamma \Delta_{z}\right)),$$

where $U^{l-1}\left(u,v\right)=F\left(u^{l-1}\left(x,y,z\right)\right)$ is the Fourier transform of the output light field in the $\left(l-1\right)$-th layer, $\Delta_{z}$ is the axial distance between the l-th layer and $\left(l-1\right)$-th layer, and $\gamma =\sqrt{1/\lambda^{2}-u^{2}-v^{2}}$.

In this study, we investigated the feasibility of using visible light as the light source for the all-optical D2NN. The He-Ne laser with 632.8 nm wavelength was selected for the networks with 5 diffraction layers in our numerical experiments. The neuron distribution of the diffractive layers was set to 200 × 200 (40,000 neurons per layer, and the size of each layer is 0.8 mm × 0.8 mm) and 400 × 400 (160,000 neurons per layer, and the size of each layer is 1.6 mm × 1.6 mm) for detection and classification tasks, respectively. The axial distance between adjacent layers, including the detection plane, was set to 10 mm. Although the diffractive angle is not large enough to achieve full connectivity in the classification task [43], a sufficient number of neurons are obtained in the diffractive layers to modulate the secondary wave field created by the previous layer, and the networks still have a considerable number of trainable connections for training.

For our experiments, we clipped the CT images into 50 × 50 pixels and resized them using nearest interpolation to 200 × 200 pixels and 400 × 400 pixels. In the training section, we set a batch size of 64 and the learning rates to 0.005 and 0.001 for pulmonary nodule detection and classification, respectively. The networks were trained for 120 epochs, and then we analyzed its inference performance on the blind test set. The results of the networks are indicated with the maximum intensity in the designed regions of the detecting plane, presenting the real-time computing results.

### 2.3. Pulmonary Nodule Detection

The network model was trained to detect the presence of nodules in CT images from the LIDC-IDRI dataset. Images were clipped around the center of each nodule and labeled as nodule regions, while images of the same size without nodules were also clipped and labeled as no-nodule regions. The number of images in both classes was balanced for training, and the dataset was divided into validation, test, and training sets in the ratio of 8:17:75.

During the training section, the propagated light amplitudes in 2 output detection regions were normalized by using $A_{i}^{\prime }=A_{i}/\left(A_{0}+A_{1}+b_{0}\right)+b_{1}\left(i=0,1\right)$, where $A_{i}$ is the sum amplitude of light field in the i-th detector, and $b_{0}$ and $b_{1}$ are 2 bias factors. The regions without nodules may have a large dark area, and the light intensity in detectors may be close to zero; thus, the factors $b_{0}$ and $b_{1}$ were applied in the normalization equation. The softmax cross-entropy loss was applied to optimize the network, as described in Equation (4) below [33]:

(4) $$L_{crossentropy}=-\left[A_{0}^{label}\log(\frac{\exp(A_{0}^{\prime })}{\exp(A_{0}^{\prime }+A_{1}^{\prime })})+A_{1}^{label}\log(\frac{\exp(A_{1}^{\prime })}{\exp(A_{0}^{\prime }+A_{1}^{\prime })})\right],$$

**Figure 2:**
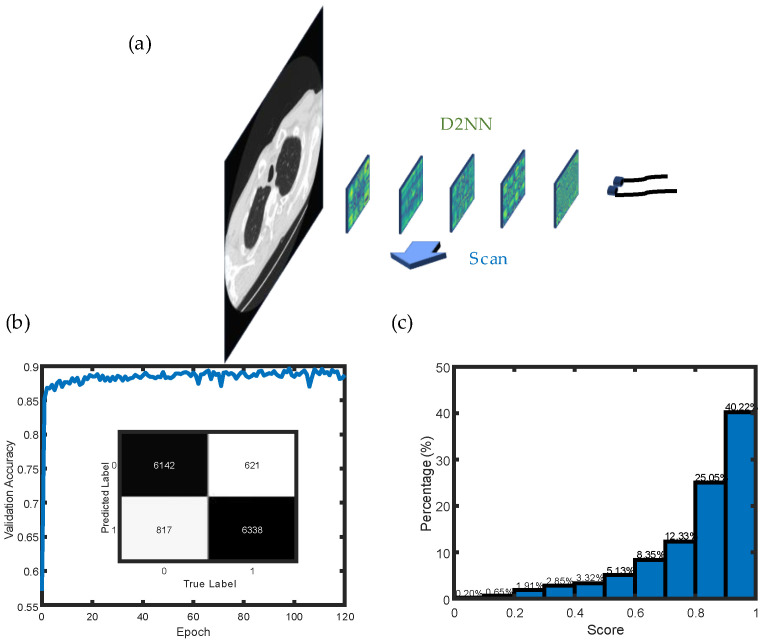
(**a**) Schematic diagram of scanning the slice of the CT image with the trained networks. (**b**) Training results of the networks and confusion matrix in test results. (**c**) Distribution of scores for nodules in the test set.

The networks were trained to classify nodules by scanning the entire CT image slices (see Figure 2). Equation (5) was applied to analyze the output of the network, obtaining the probability of nodules’ existence as the score:

(5) $$score=\frac{A_{i}^{\prime }}{A_{0}^{\prime }+A_{1}^{\prime }}\left(i=0,1\right).$$

### 2.4. Pulmonary Nodule Classification

The location and classification of the nodules are provided in XML files, which divide the nodules into 5 classes (labeled 1–5). Benign nodules were labeled as “1” or “2”, while malignant nodules were labeled as “4” or “5”. Nodules labeled as “3” were discarded. To prepare the images for training, the images were clipped to a size of 50 × 50 pixels, using the same method as mentioned in Section 2.3. The cases were also divided into validation, test, and training sets, with a ratio of 8:17:75, respectively. In addition, traditional data augmentation methods, such as rotating and flipping the images, were utilized to increase the number of images in the training set.

During the training process, the intensities of the 2 detectors in the output planes were also evaluated by using factor α, as follows:

(6) $$A_{i}^{\prime }=\alpha \frac{A_{i}}{\max(A_{0},A_{1})}\left(i=0,1\right),$$

where $A_{0}$ and $A_{1}$ are the sum amplitudes in the 2 detectors’ regions. The mean square error loss function (7) was applied to optimize the network as follows [23]:

(7) $$L_{mse}={\left(A_{0}^{\prime }-\alpha A_{0}^{label}\right)}^{2}+{\left(A_{1}^{\prime }-\alpha A_{1}^{label}\right)}^{2}.$$

## 3. Results

**Figure 3:**
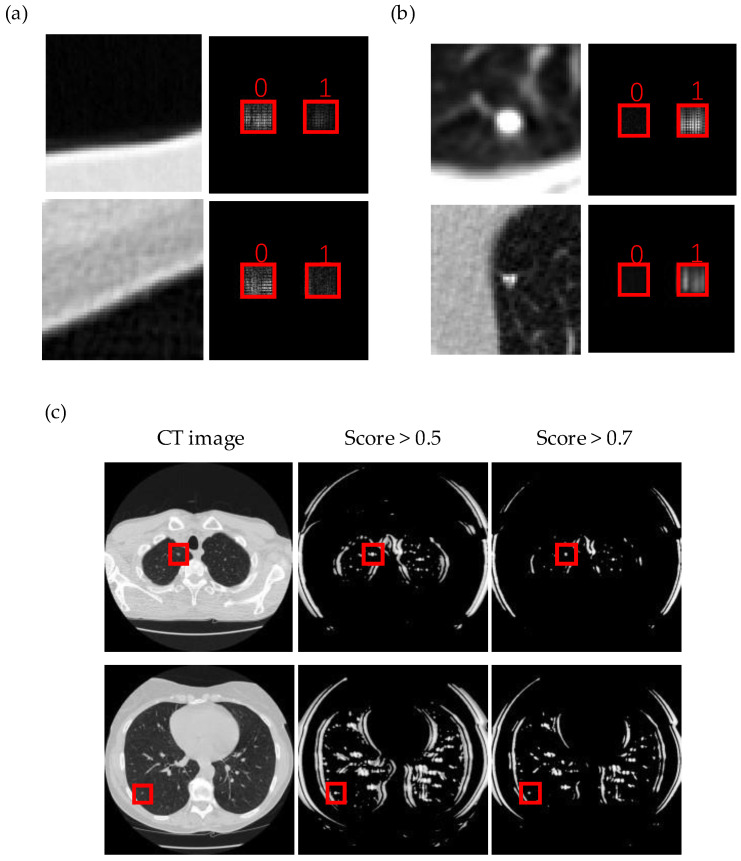
(**a**,**b**) Amplitude of the light field in forward inference results for negative and positive samples in the test set, respectively. The left image represents the input image, and the right image shows the amplitude of the light field in the two detectors’ regions (as marked in red squares) in the output plane. The number “0” and “1” present the possibilities of negative and positive class, respectively. (**c**) Scan result of CT images. The white points on the images represent the regions that possibly have nodules, while the black points on the images represent the regions that have almost no nodules. The red square region shows the center of nodules in ground truth.

Figure 2a,b present the training section of the networks and the accuracy of the network on the validation set converging after a few epochs, respectively. The detailed results are presented in the first two rows in Table 1. The networks’ accuracy in the test set is 89.67% in the two-class classification, and the recall rate reaches 91.08%. The dataset can also be split into 10 parts with 10-fold cross validation, indicating that the mean accuracy in 10 folds is 89.72%, which is close to the performance in the test set. The score of each nodule in the test set was calculated to determine the existence possibility of the nodules. Figure 2c shows the distribution of scores, indicating that most nodules have a score higher than 0.7. In this case, the threshold of the score can be set higher than 0.5, and, at the same time, most of the regions can be detected with a correct result. The outputs of the networks were obtained from two detectors in the detection plane by comparing the amplitude of the light. In Figure 3a,b, the real-time inference results are shown, and the classification results can be clearly obtained by simply comparing the intensity in two detections directly.

**Figure 4:**
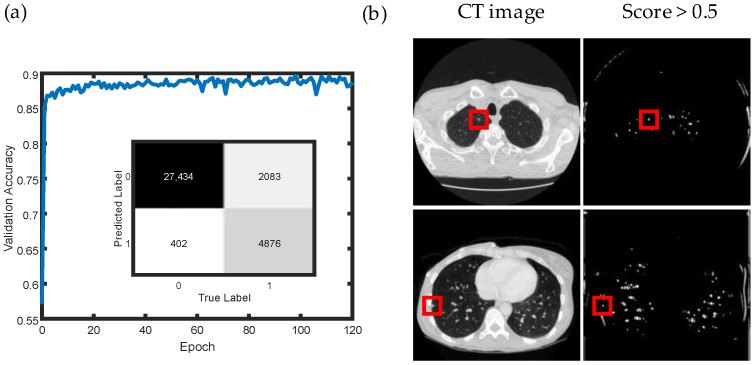
(**a**) Training section and confusion matrix in the test set of the dataset, whose ratio of nodules to non-nodules is 1:4. (**b**) Scan results of CT images. The white points on the images represent the regions that possibly have nodules, while the black points on the images represent the regions that have almost no nodules. The red square region shows the center of nodules in ground truth.

The trained networks were also applied to scan the CT image slices to search and detect nodules. The existence probability of the nodules was determined by the score of the clipped CT images, and a threshold was selected to assess the presence of nodules. Although there are many false-positive points in the results, almost all the nodules could be detected based on the networks’ recall as shown in Figure 3c. Meanwhile, increasing the threshold can discard many false-positive points. However, the recall rate also reduced to 77.60% with a threshold of 0.7. Additionally, many regions without nodules are not included in the dataset, which also further influences the result. To balance the difference ratio of images with and without nodules, the ratio was set to 1:4 to train and test the networks again. The training results and the confusion matrix are shown in Figure 4a, indicating the classification ability of the networks. The last two rows in Table 1 provide the detailed results of this trained network. The average accuracy in 10-fold cross validation is 92.49%, which is close to the accuracy in the test set (92.86%). The scan result is shown in Figure 4b, and the false-positive points are much less than before. However, the recall rate is also reduced to 70.07%, meaning that just 70.07% of the nodules are detected in the test set. In this case, both the threshold setting and the ratio of positive and negative samples influenced the result of the networks’ performance.

**Figure 5:**
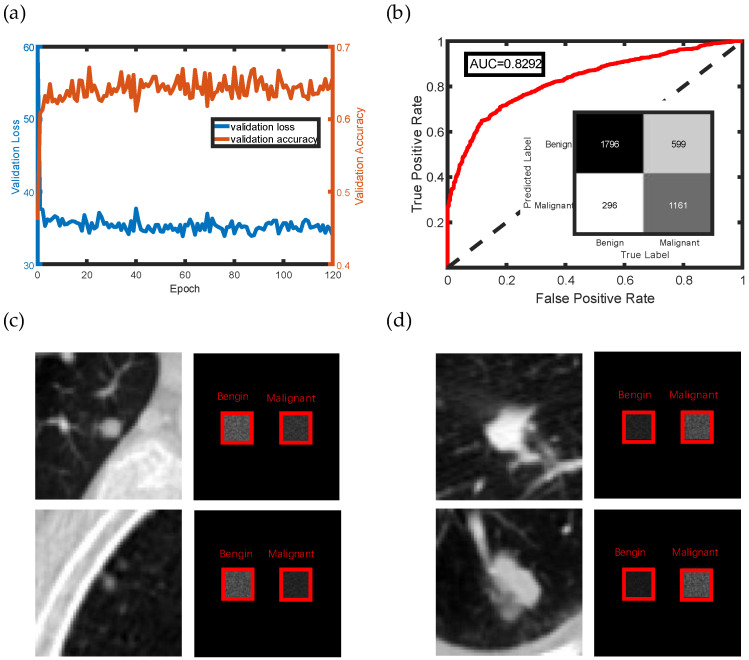
(**a**) Training results of nodule classification. (**b**) Confusion matrix and receiver operating characteristic (ROC) curve for the inference result in the test set. (**c**,**d**) Amplitude of the light field in forward inference of benign and malignant nodules, respectively. The left image represents the input image, and the right image shows the amplitude of two detectors’ regions in the output plane.

The networks were also used to classify nodules into benign and malignant categories. Figure 5a shows the training results, where the loss decreases quickly, and the network converges after a few epochs of training. Table 2 shows the performance of the trained network in the validation and test sets. The accuracy in the test set is 76.77%, and the recall rate reaches 65.97%, which is slightly different from that of the validation set. The reason may be that some difficult classified singular malignant nodules, were split in the validation set and there was not enough data to validate the performance of the trained network. Furthermore, 10-fold cross validation was performed, showing that the max accuracy reaches 79.43% with a mean accuracy of 74.59%. The confusion matrix and ROC curve in the test set are shown in Figure 5b, with an AUC of 0.8292, indicating the credible classification result. The field distribution is shown in Figure 5c,d, when the images were inferred on the networks, with the left detector representing benign nodules and the right detector representing malignant nodules. The real-time output is the label of the region with the highest intensity.

## 4. Discussion

In this paper, we present the model of an all-optical deep diffractive neural network, which was trained and employed to perform nodule detection and classification tasks using the LIDC-IDRI dataset. The nodule detection task involved determining whether nodules were present or not, which was achieved with an accuracy of 87.78% and a recall rate of 90.47%. The trained networks were further used to scan CT image slices to detect nodules. Although the recall ratio in this study is similar to that of others, as shown in Table 3, it should be noted that traditional deep learning methods considered the whole size of CT images in the training section while our network only focuses on the partial section of CT images and considers only the centers of nodules as the targets. This explains why many false-positive points were detected in this study. Despite the performance of our all-optical network being slightly poorer than that of other computer-based methods, the classification of benign and malignant nodules achieved an accuracy of 76.77%, with an AUC of 0.8292, as shown in Table 4. The performance of the all-optical network could be improved by incorporating more non-linear computing sections. Overall, the simulation results demonstrate the potential of all-optical neural networks in real-time processing of medical images for aided diagnosis.

On the other hand, the network can be fabricated in experiments using optical devices, and its inference process can achieve speeds similar to light flight [23]. The network can be divided into three parts: the light source, optical diffractive layers, and detectors. The light source is the input of the network, while the optical diffractive layers modulate the light field to perform designed computation. The optical diffractive layers can be fabricated using a 3D-printed technique [23], multi-step photolithography–etching method [43], or metasurface technique [44]. The number of modulation units (trained parameters) does not affect the inference speed of the network, as the speed of light is constant. The detectors collect the final intensity of light, and once the input image is loaded, the distribution of light intensity can be directly seen at the detectors immediately, which represents the result of the network processing. Since the all-optical network has a forward inference speed similar to light, it has been reported in many fields [34,35,36,37,49,50,51].

In addition, the computation power of all-optical networks is currently limited due to the lack of non-linear computing. However, integration with optical non-linear materials, such as magneto-optical traps [52] and photo-refractive crystals [53], provides the possibility to enhance the computation power and further improve the precision of nodule detection and classification. Moreover, as manufacturing processes continue to develop, it may be possible to fabricate an integrated device that can address both nodule detection and classification, provided that non-linear materials can be incorporated into the device. Hopefully, this approach of using all-optical fast computation devices in medical image real-time processing for aided diagnosis will soon become a reality.

## Figures and Tables

**Table 1:** Nodule Detection Task Results in Validation and Test Sets.

Work	Accuracy (%)	Recall (Sensitivity) (%)	Precision (%)	F1 Score	MMC
Trained with 1:1 ratio (validation set)	89.54	90.96	88.44	0.8968	0.7911
Trained with 1:1 ratio (test set)	89.67	91.08	88.58	0.8981	0.7937
Trained with 1:4 ratio (validation set)	92.67	69.66	91.68	0.7917	0.7586
Trained with 1:4 ratio (test set)	92.86	70.07	79.68	0.7218	0.8585

MMC: Matthews Correlation Coefficient.

**Table 2:** Nodule Classification Task Results in Validation and Test Sets.

Work	Accuracy (%)	Recall (Sensitivity) (%)	Precision (%)	F1 Score	MMC
Validation set	67.13	51.35	77.08	0.6164	0.3706
Test set	76.77	65.97	79.68	0.7218	0.5323

MMC: Matthews Correlation Coefficient.

**Table 3:** Comparison with Other Studies in Nodule Detection Task.

Study	Recall (Sensitivity) (%)	Runtime
Ali et al. [38]	58.9	DPPU
Harsono et al. [39]	94.12	DPPU
Cao et al. [40]	92.5	DPPU
Ours trained with 1:1 ratio	91.08	Real time
Ours trained with 1:4 ratio	70.07	Real time

DPPU: Depend on the performance of processing unit.

**Table 4:** Comparison with Other Studies in Nodule Classification Task.

Study	Accuracy (%)	Recall (Sensitivity) (%)	Specificity (%)	AUC	Runtime
Song et al. [41]	82.59	83.96	81.35	0.884	DPPU
Nibali et al. [47]	89.90	91.07	88.64	0.9459	DPPU
Zhao et al. [48]	82.2	NA	NA	0.877	DPPU
Apostolopoulos et al. [42]	92.07	89.35	94.80	0.9208	DPPU
Ours	76.77	65.97	85.85	0.8292	Real time

DPPU: Depend on the performance of processing unit.

## Data Availability

Data underlying the results presented in this paper are not publicly available at this time but may be obtained from the authors upon reasonable request.
